# Hypoxia Inducible Factor 1α Inhibits the Expression of Immunosuppressive Tryptophan-2,3-Dioxygenase in Glioblastoma

**DOI:** 10.3389/fimmu.2019.02762

**Published:** 2019-12-04

**Authors:** Soumya R. Mohapatra, Ahmed Sadik, Lars-Oliver Tykocinski, Jørn Dietze, Gernot Poschet, Ines Heiland, Christiane A. Opitz

**Affiliations:** ^1^DKTK Brain Cancer Metabolism Group, German Cancer Research Center (DKFZ), Heidelberg, Germany; ^2^Faculty of Bioscience, Heidelberg University, Heidelberg, Germany; ^3^Division of Rheumatology, Department of Medicine V, University Hospital of Heidelberg, Heidelberg, Germany; ^4^Department of Arctic and Marine Biology, UiT The Arctic University of Norway, Tromsø, Norway; ^5^Centre for Organismal Studies (COS), University of Heidelberg, Heidelberg, Germany; ^6^Neurology Clinic and National Center for Tumor Diseases, University Hospital of Heidelberg, Heidelberg, Germany

**Keywords:** hypoxia, HIF1α, tryptophan, TDO2, immunosuppression

## Abstract

Abnormal circulation in solid tumors results in hypoxia, which modulates both tumor intrinsic malignant properties as well as anti-tumor immune responses. Given the importance of hypoxia in glioblastoma (GBM) biology and particularly in shaping anti-tumor immunity, we analyzed which immunomodulatory genes are differentially regulated in response to hypoxia in GBM cells. Gene expression analyses identified the immunosuppressive enzyme tryptophan-2,3-dioxygenase (TDO2) as the second most downregulated gene in GBM cells cultured under hypoxic conditions. TDO2 catalyses the oxidation of tryptophan to N-formyl kynurenine, which is the first and rate-limiting step of Trp degradation along the kynurenine pathway (KP). In multiple GBM cell lines hypoxia reduced TDO2 expression both at mRNA and protein levels. The downregulation of TDO2 through hypoxia was reversible as re-oxygenation rescued TDO2 expression. Computational modeling of tryptophan metabolism predicted reduced flux through the KP and lower intracellular concentrations of kynurenine and its downstream metabolite 3-hydroxyanthranilic acid under hypoxia. Metabolic measurements confirmed the predicted changes, thus demonstrating the ability of the mathematical model to infer intracellular tryptophan metabolite concentrations. Moreover, we identified hypoxia inducible factor 1α (HIF1α) to regulate TDO2 expression under hypoxic conditions, as the HIF1α-stabilizing agents dimethyloxalylglycine (DMOG) and cobalt chloride reduced TDO2 expression. Knockdown of HIF1α restored the expression of TDO2 upon cobalt chloride treatment, confirming that HIF1α controls TDO2 expression. To investigate the immunoregulatory effects of this novel mechanism of TDO2 regulation, we co-cultured isolated T cells with TDO2-expressing GBM cells under normoxic and hypoxic conditions. Under normoxia TDO2-expressing GBM cells suppressed T cell proliferation, while hypoxia restored the proliferation of the T cells, likely due to the reduction in kynurenine levels produced by the GBM cells. Taken together, our data suggest that the regulation of TDO2 expression by HIF1α may be involved in modulating anti-tumor immunity in GBM.

## Introduction

More than 60 years ago, Thomlinson and Gray postulated the occurrence of hypoxic regions in solid tumors (1). Initial interest in studying hypoxia in tumors was due to the realization that hypoxic cells are more resistant to radiotherapy (2) resulting in adverse clinical outcomes for the patients. Measurements of intra-tumoral oxygen levels revealed a highly heterogeneous hypoxic landscape within a tumor. The oxygen concentration in moderately hypoxic regions was determined to be ~1% oxygen (O_2_), while in severely hypoxic tumor regions it fell below 1% O_2_ (3).

Cells under hypoxic conditions are known to shut down non-essential processes by chromatin modifications and a global downregulation of gene expression (4), while simultaneously genes needed for cell survival under oxygen limitation are upregulated through hypoxia inducible transcription factors (HIFs) (5). The most well-known HIF family member HIF1α is degraded under normoxic conditions by the action of prolyl-hydroxylase (PHD) enzymes. However, under hypoxic conditions PHD function is inhibited, thus stabilizing HIF1α and activating the expression of its target genes (6). Furthermore, HIF1α can also inhibit the expression of genes during hypoxia (7).

Similar to other solid tumors, the most aggressive primary brain tumor, glioblastoma multiforme (GBM), is prone to severe hypoxia. GBM are characterized by the presence of necrotic foci and surrounding severely hypoxic pseudopalisades consisting of outwardly migrating GBM cells trying to escape the core hypoxic regions (8). Measurements of O_2_ levels in tumors from 14 GBM patients revealed a median O_2_ level of 0.7% (9). Bio-availability of oxygen to a tumor cell in a solid tumor depends on a number of factors such as the distance of the tumor cell from the nearest blood vessel and diminished blood supply due to the abnormal vasculature found in tumors (10, 11). Irrespective of the factors causing hypoxic stress, hypoxia has been shown to drive established hallmarks of cancer progression in GBM such as inhibition of cell death (12), induction of angiogenesis (13), activation of endothelial to mesenchymal transition (14), modulation of cellular metabolism (15), and tumor immune escape (16).

Of note, hypoxic regions of solid tumors often harbor a large number of immunosuppressive cells, which inhibit anti-tumor immune responses (17). Tumor hypoxia in GBM has been shown to exert immune suppression by activation of regulatory T cells (Tregs) (18). HIF1α-mediated gene regulation is involved in promoting hypoxic suppression of anti-tumor immunity (19, 20). For instance, HIF1α induces the expression of the inhibitory immune checkpoint regulator programmed death-ligand 1 (PD-L1), which facilitates the suppression of anti-tumor immune effects (21, 22). Furthermore, hypoxia also obstructs anti-tumor immunity by reduction of tumor cell MHC presentation and the tumor cell expression of chemokines essential for immune cell infiltration (23).

In light of the important role played by hypoxia in GBM biology and particularly in modulating anti-tumor immune responses, we analyzed GBM cells for genes involved in the regulation of anti-tumor immunity that are differentially regulated upon hypoxia. We find that hypoxia significantly reduces the expression of the immunosuppressive enzyme tryptophan-2,3-dioxygenase (TDO2). TDO2 catalyses the first step of tryptophan (Trp) catabolism along the kynurenine pathway (KP) and is known to play an important role in GBM as it promotes tumor cell motility and suppresses anti-tumor immune responses via production of Trp metabolities that activate the aryl hydrocarbon receptor (AHR) (24).

## Materials and Methods

### Cell Culture

Human GBM cell lines A172, U-87 MG, and LN-18 were obtained from ATCC. Cells were cultivated in DMEM (Gibco) containing 10% FBS (Gibco), 2 mM Glutamine (Gibco), 1 mM Sodium Pyruvate (Gibco), 100 μg/ml Streptomycin, and 100 U/ml Penicillin (Gibco). Cells were authenticated by Multiplex Cell Authentication service (Multiplexion GmbH) and were routinely monitored using the Venor® GeM Classic mycoplasma detection kit (Minerva Biolabs). Twenty-four hours after cell seeding a medium change was done following which, various treatments were carried out. Unless stated otherwise, cells were normally cultivated at normoxic conditions i.e., 18.6% O_2_ concentration (conc.) (25) in a SANYO MCO-18AIC incubator with 5% CO_2_ and at 37°C.

### Long Term Hypoxic Exposure

A172, U-87 MG, and LN-18 cells in T25 flasks containing 5 ml medium, were subjected to either normoxic or hypoxic conditions for 3, 5, 8, and 10 days. For hypoxic conditions (i.e., 1% O_2_) cells were placed in the Labotect incubator C42. For each time point, cells were seeded in duplicates; one of the flasks was incubated in normoxic conditions and served as a control for the other flask incubated under hypoxic conditions. At the indicated time points, 1 ml culture supernatant from each treatment was harvested and used for metabolic measurements. Subsequently, the cells were harvested by trypsinization in 1.5 ml PBS. The cell count of the harvested cells was measured using 10 μl of cell suspension and Trypan Blue dye (Gibco) in a 1:1 ratio with an automated Cell Counter (Countess, Invitrogen). The remaining cells were further processed for either RNA, protein or intracellular metabolite extraction.

### Treatment With Hypoxia Mimetics

HIF1α protein stabilization was attained by using the hypoxia mimetic compounds dimethyloxalylglycine (DMOG) and cobalt chloride (CoCl_2_). DMOG was obtained from Frontier Scientific Inc. and reconstituted in 100% ethanol (Sigma). Cells were treated such that the final DMOG concentrations of 0.5, 1, 2, and 3 mM were obtained according to the treatment specifications. EtOH was used as carrier control. A second hypoxia mimetic agent, cobalt chloride (CoCl_2_) was obtained from Sigma and reconstituted in ddH_2_O. Cells were treated for 24 h such that a final treatment concentration of 100, 150, 200, 250, and 300 μM of CoCl_2_ was obtained.

### RNA Isolation and Quantitative (q)RT-PCR

Total RNA was isolated using the RNAeasy Mini Kit (Qiagen). cDNA was reverse transcribed from 1 μg total RNA using the High Capacity cDNA reverse transcriptase kit (Applied Biosystems). cDNA amplification was performed using the SYBR Select Master Mix (Thermo Fisher Scientific) during the (q)RT-PCR on a StepOnePlus real-time PCR system (Applied Biosystems). For all (q)RT-PCR measurements 18s RNA was used as a housekeeping gene for normalization. (q)RT-PCR primers for a gene were designed using Primer Blast (NCBI), such that on the genomic DNA at least one intron separated the forward and reverse primers.

Primer sequences used:

**Table:** 

TDO2	Forward	5′-CAAATCCTCTGGGAGTTGGA-3′
	Reverse	5′-GTCCAAGGCTGTCATCGTCT-3′
18s RNA	Forward	5′-GATGGGCGGCGGAAAATAG-3′
	Reverse	5′-GCGTGGATTCTGCATAATGGT-3′
NDRG1	Forward	5′-TCAAGATGGCGGACTGTG-3′
	Reverse	5′-GAAGGCCTCAGCGAGCTT-3′

### Protein Isolation and Western Blots

Total protein was harvested using ice-cold RIPA lysis buffer (1% IGEPAL/NP40, 12 mM sodium-deoxycholate, 3.5 mM sodium dodecyl sulfate (SDS) supplemented with a protease and phosphatase inhibitor (Roche/Sigma-Aldrich). The Bradford protein assay (Biorad) was employed for total protein content measurement and subsequent normalization between samples across one experiment. Protein samples were separated using a 10% SDS-PAGE gel and transferred onto an activated 0.45 μm PVDF membrane (Sigma-Aldrich), subsequently the membrane was blocked with 5% BSA for 30 min and incubated with primary antibodies overnight. The primary antibodies were used in 1:1000 dilution for mouse anti-human TDO2 (#TA504730, Origene), rabbit anti-human NDRG1 (#HPA006881, Sigma), and rabbit anti-human Tubulin (#ab108629, Abcam plc.). Membranes were subsequently incubated for 1 h with 1:5000 diluted HRP-conjugated secondary antibodies (anti-rabbit ab.: #GENA9340-1M, anti-mouse ab.: #GENXA931, both from GE Healthcare). Either Pierce® ECL Western Blotting Substrate or SuperSignal® West Femto Maximum Sensitivity Substrate (both Thermo Scientific) were used to generate the signals, which were captured either on an autoradiography film (Amersham Hyperfilm, GE Healthcare) or on the ChemiDoc XRS+ (Bio-Rad Laboratories) using Image Lab Software 5.2.1.

### siRNA-Mediated HIF1α Knockdown

siRNA stocks (20 μM) were prepared by reconstituting ON-TARGETplus Human SMARTPOOL siRNA reagent targeting HIF1α (Dharmacon) in sterile PBS in accordance to the manufacturer's recommended ratio. ON-TARGETplus Non-targeting Pool siRNA (Dharmacon) was used as a control. siRNA transfection mix was prepared using siRNA stocks and Lipofectamine RNAiMAX (Thermo Fisher Scientific) according to the manufacturer's protocol. Cells were treated with the transfection mix for 24 h, which was followed by a fresh medium change and incubation with cobalt chloride for 24 h before harvest.

### High Performance Liquid Chromatography (HPLC)

For Trp and Kyn measurements (**Figures 2A,B**) in cell culture supernatants, 72% trichloroacetic acid (Sigma-Aldrich) was added in a ratio of 162.8 μl per 1 ml of supernatant for protein precipitation. Samples were then centrifuged at full speed for 12 min, following which the supernatants were analyzed in a Dionex Ultimate® 3000 uHPLC (Thermo Scientific, Waltham, MA, USA) by chromatographic separation. An Accucore™ aQ column (Thermo Scientific™) with 2.6 μm particle size with a gradient mobile phase consisting of 0.1% trifluoroacetic acid (TFA) in water (A) and 0.1% TFA in acetonitrile (B) was utilized for separation of Trp and Kyn, which were detected at UV emission spectra of 280 and 365 nm, respectively. The Chromeleon™ 7.2 Chromatography Data System (Thermo Scientific™ Dionex™) was used for data analysis.

For intracellular analyses of Trp and Trp-derived compounds (**Figure 2C**), the samples were rapidly frozen in liquid nitrogen following trypsinization and pelleting of harvested cells. Subsequently, metabolites were extracted with 0.1 ml 6% perchloric acid per million of cells in an ultrasonic ice-bath for 10 min. For analyses of extracellular content, supernatants were mixed with an equal volume of 12% perchloric acid and incubated on ice for 10 min. Prior analysis, samples were centrifuged for 10 min at 4°C and 16.400 g to precipitate proteins and to remove remaining cell debris. Metabolites were separated by reversed phase chromatography on an Acquity HSS T3 column (100 × 2.1 mm, 1.7 μm, Waters) connected to an Acquity H-class UPLC system (Waters). The column was heated to 37°C and equilibrated with 5 column volumes of 100% solvent A (20 mM sodium acetate, 3 mM zinc acetate, pH 6) at a flow rate of 0.55 ml min^−1^. Clear separation of Trp and Trp-derived compounds was achieved by increasing the concentration of solvent B (Acetonitrile) in solvent A as follows: 4 min 0% B, 10 min 5% B, 13 min 15% B, 15 min 25% B, and return to 0% B in 3 min. Trp, 3HAA, KynA and tryptamine were detected by fluorescence (Acquity FLR detector, Waters, excitation: 254 nm, emission: 401 nm). Kyn and OH-Kyn were determined by simultaneous recording of absorption at 365 nm (Acquity PDA detector, Waters). For quantification, ultrapure standards were used (Sigma). Data acquisition and processing was performed with the Empower3 software suite (Waters).

### Microarray Analysis

RNA was harvested after 5 days from the control and hypoxic cells, using the RNAeasy Mini Kit (Qiagen). Labeled ss-cDNA was generated from 100 ng total RNA using the Affymetrix WT PLUS Reagent Kit, as per the manufacturer's instructions. Subsequently, 5.5 μg of fragmented and labeled ss-cDNA were hybridized for 17 h at 45°C on Affymetrix Human Gene 2.0 ST chip following the manufacturer's instructions. The Affymetrix GeneChip® Scanner 3000 was used for scanning the hybridized chips according to the GeneChip® Expression Wash, Stain and Scan Manual for Cartridge Arrays (P/N 702731). The Raw CEL files were imported from disk followed by RMA normalization and summarization using the *oligo* package and were annotated at the probeset level using NetAffx (26). Differential gene expression was conducted by fitting a linear model and estimating a moderated *t*-statistic followed by eBayes adjustment as described in the *limma* package (27, 28). All analyses were run in R, version 3.4.4 (https://cran.r-project.org/) and Bioconductor version 3.6 (https://bioconductor.org/). All graphical representations were generated using *ggplot2, ggpubr*, and *RcolorBrewer*. All datasets have been made publicly available in the Gene Expression Omnibus (GEO) repository under accession number GSE138535.

### Modeling of Trp Metabolism

To simulate Trp metabolism in A172 cells, we employed the previously published comprehensive kinetic model of Trp metabolism (29). Microarray data previously generated for A172 cells exposed to normoxia or hypoxia for 5 days, were integrated into the Trp model using SBMLmod as described previously (30). No transporters and no external metabolites were used. Instead the concentration of cellular Trp was set to the measured intracellular concentration, assuming a cell volume of 10 pL. Steady-state concentrations and fluxes were calculated using the steady state task of COPASI 4.26 (31).

### Proliferation Measurements in T Cell Co-cultures

The functional characterization of PBMC from healthy donors was approved by the Ethics Committee of the University of Heidelberg. PBMC isolation was carried by density-gradient centrifugation and T cells were enriched using the MojoSort Human CD4 T cell isolation kit (Biolegend) according to the manufacturer's instructions. T cells were cultured in 96 well plates either alone or in a co-culture with A172 cells in RPMI1640 (Thermo Fisher Scientific) containing 10% FCS in the presence of 5 μg/ml phytohaemagglutinin (PHA) (Sigma) and 1 U/μl rhIL-2 (Novartis). All cultures were performed in duplicate sets, one set under normoxia and the second under hypoxia. A glove box (Coylab) and an incubator (Heracell 150i, Thermo Fisher Scientific) with oxygen level regulation were used for ensuring continued hypoxia conditions during all steps of culturing. PKH26 (Sigma-Aldrich) was used to label T cells prior to culture. After 6 days of culturing, PKH26 mean fluorescence intensity (MFI) was measured by flow cytometry (BD FACSCanto II (BD Biosciences). The degree of reduction in PKH26 fluorescence intensity reflected the number of cell divisions undergone by the cells. Suppression of T cell proliferation by hypoxia was measured by calculating the ratio between the PKH26 MFI of T cell co-cultured with A172 cells and the PKH26 MFI of T cells cultured alone, for both normoxia and hypoxia culture conditions.

### Statistical Analysis

GraphPad Prism v5.04 (GraphPad Software Inc.) was used for performing statistical analysis. For single comparisons between two datasets, two-tailed unpaired Student's *t*-test was utilized. Rank sum analysis by the Mann–Whitney *U*-test was carried out wherever necessary. For multiple comparisions, one-way ANOVA with Tukey's multiple comparisons test was employed. Data was collected from at least three independent experiments. All data are plotted as mean ± SEM, unless stated otherwise. Differences with a *p* ≤ 0.05 were considered to be statistically significant (ns: not significant i.e., *p* > 0.05; ^*^p ≤ 0.05; ^**^p ≤ 0.01; ^***^p ≤ 0.001; ^****^p ≤ 0.0001).

## Results

### TDO2 Expression Is Suppressed Under Hypoxia

**Figure 1:**
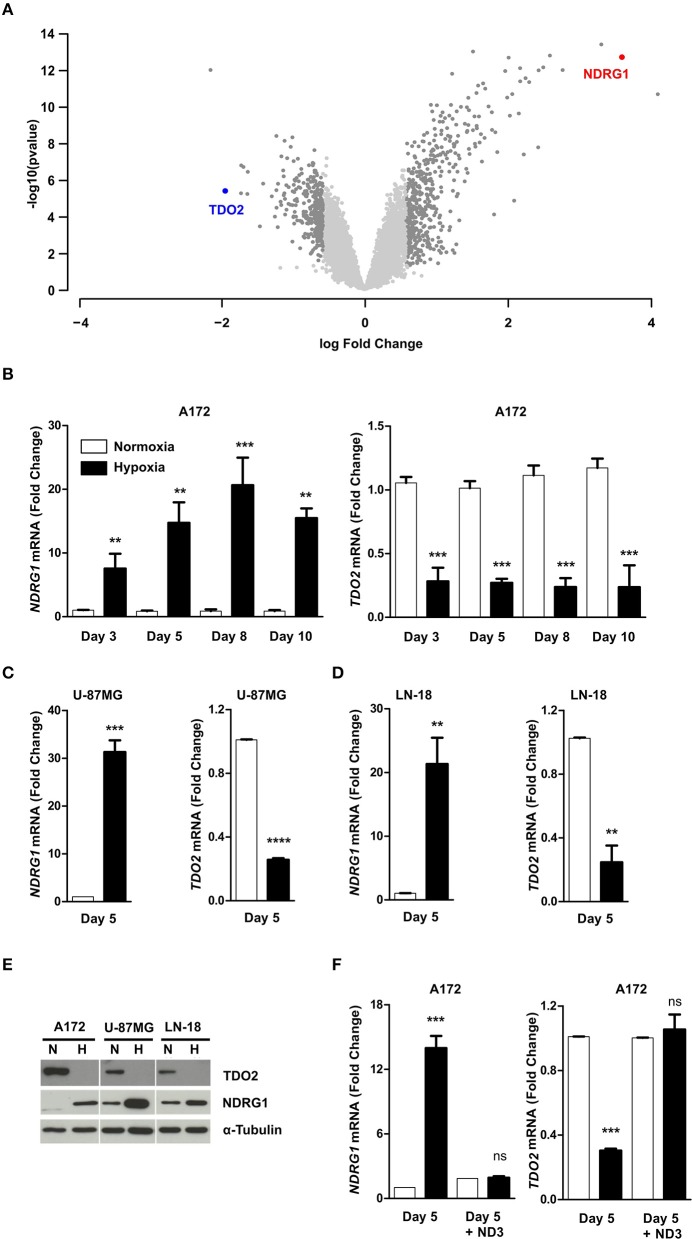
Hypoxia reversibly downregulates tryptophan-2,3-dioxygenase (TDO2) expression in GBM cells. **(A)** Volcano plot showing differentially regulated genes in A172 cells upon exposure to 5 days of hypoxia compared to 5 days normoxic controls. **(B)** qRT-PCR analysis of NDRG1 (left) and TDO2 (right) mRNA expression in A172 cells after 3, 5, 8, or 10 days of exposure to either normoxia (white) or hypoxia (back). **(C)** qRT-PCR analysis of NDRG1 (left) and TDO2 (right) mRNA expression in U-87MG cells after 5 days of either normoxia (white) or hypoxia (black) exposure. **(D)** qRT-PCR analysis of NDRG1 (left) and TDO2 (right) mRNA expression in LN-18 cells after 5 days of either normoxia or hypoxia. **(E)** Western blot analysis of TDO2 and NDRG1 protein expression in A172, U-87MG, and LN-18 cells subsequent to 5 days normoxia or hypoxia. α-Tubulin protein expression was used as a loading control. **(F)** NDRG1 (left) and TDO2 (right) mRNA expression in A172 cells analyzed by qRT-PCR after exposure to hypoxia for 5 days followed by re-oxygenation for 3 days under normoxic conditions (ND3). Data from at least three independent experiments are expressed as mean ± S.E.M. Statistical significance was assumed at *p* < 0.05 (**p < 0.01, ***p <0.001, ****p ≤ 0.0001). n.s., not significant.

To investigate if hypoxia differentially regulates genes that play a role in anti-tumor immune responses in GBM cells, we performed microarray analysis of A172 GBM cells exposed to 5 days of hypoxia (1% O_2_) as compared to cells cultured in normoxia (18.6% O_2_) (GSE138535). Analysis of the microarray data revealed tryptophan-2,3-dioxygenase (TDO2) to be the second most downregulated gene under hypoxia (Figure 1A, Supplementary Table 1). TDO2 is an immunosuppressive enzyme, whose metabolic products have been shown to modulate anti-tumor immune responses by inhibition of T cell proliferation as well as induction of apoptosis in T cells (32, 33). Apart from TDO2, other immune-regulatory genes, such as TLR3 and CCL2 were also strongly downregulated under hypoxia (Supplementary Table 1). However, in the present study we focussed our attention on TDO2, the strongest differentially regulated gene candidate among the genes with known effects on immune responses. TDO2 integrates molecular O_2_ into Trp to generate formyl-kynurenine, which is further converted to kynurenine (34). Therefore, reduced O_2_ concentrations under hypoxia would be expected to affect the enzymatic activity of TDO2, however our microarray data revealed that also the expression of TDO2 may be reduced upon hypoxia in GBM cells.

To validate the results of the microarray, we next performed qRT-PCR measurements. To test for the presence of hypoxia we assayed N-myc downstream regulated 1 (NDRG1), a gene known to be upregulated under hypoxia in GBM (35, 36), as a surrogate hypoxia marker that was also significantly upregulated by hypoxia in the microarray (Supplementary Table 1). Analysis of mRNA transcript levels in A172 cells exposed to hypoxic conditions for different durations, confirmed the presence of hypoxia as NDRG1 was significantly upregulated (Figure 1B, left). Further, the qRT-PCR measurements confirmed the result of the microarray analysis, as a significant reduction in TDO2 mRNA levels was observed at all-time points upon hypoxic exposure (Figure 1B, right). Downregulation of TDO2 mRNA in response to hypoxia was not limited to A172 cells, but was also observed in U-87 MG and LN-18 GBM cells (Figures 1C,D). Western blot analysis of all three GBM cell lines exposed to either normoxia or hypoxia revealed that TDO2 protein expression was reduced under hypoxia, while expression of the hypoxia surrogate marker NDRG1 was increased (Figure 1E). To investigate whether the hypoxic downregulation of TDO2 expression can be restored by re-oxygenation, we subjected A172 cells to 3 days of normoxia after 5 days of hypoxia. Re-oxygenation completely restored the expression of TDO2 (Figure 1F), indicating that the observed hypoxic downregulation of TDO2 is reversible.

### Hypoxia-Mediated TDO2 Downregulation Reduces Flux Through the KP

**Figure 2:**
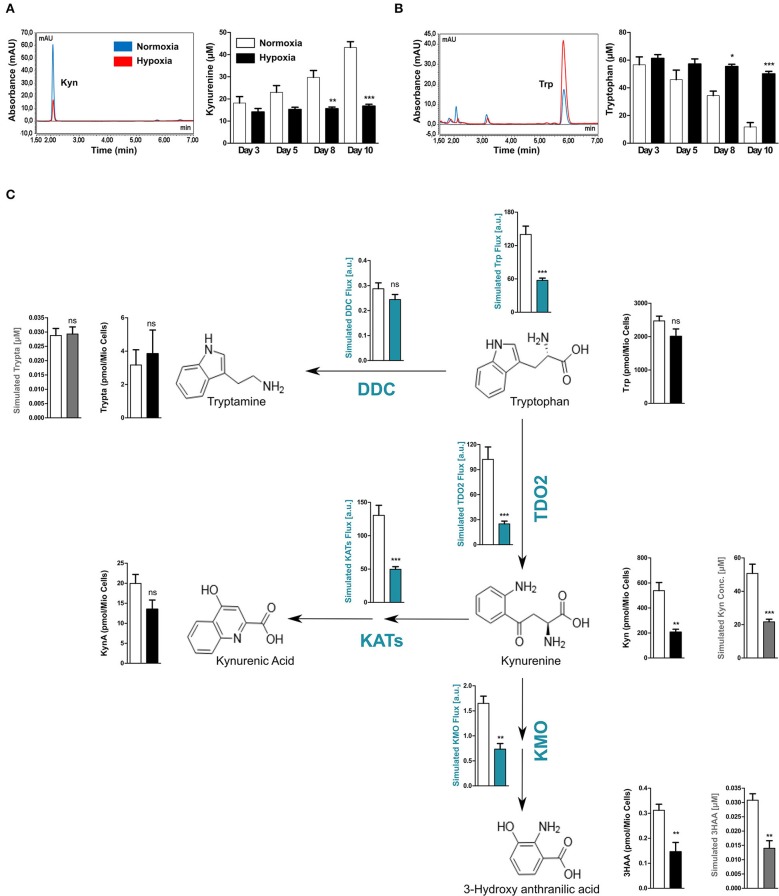
Reduced TDO2 expression leads to reduced Trp flux through the KP. **(A)** uHPLC chromatogram (left) showing Kyn measured in supernatants of A172 cells exposed to 5 days of either normoxia (blue) or hypoxia (red). Quantification of Kyn measurements (right) in supernatants of A172 cells cultured either under normoxia (white) or hypoxia (black) for 3, 5, 8, or 10 days. **(B)** uHPLC chromatogram (left) showing Trp measured in supernatants of A172 cells exposed to 5 days of either normoxia (blue) or hypoxia (red). Quantification of Trp (right) in A172 cell supernatants cultured either under normoxia (white) or hypoxia (black) for 3, 5, 8, or 10 days. **(C)** Scheme depicting the most prominent changes in the flux through the Trp degradation pathway upon exposure to hypoxia (blue plots). Microarray data from A172 GBM cells upon 5 days of hypoxia exposure was integrated into a computational model of Trp metabolism to calculate the fluxes through different enzymes and the general flux through the entire pathway (blue plots) as well as to predict the intracellular metabolite concentrations (gray plots). These intracellular predictions were validated by measurements of the intracellular concentrations (black plots). Data from at least three independent experiments are expressed as mean ± S.E.M. Statistical significance is assumed at *p* < 0.05 (*p < 0.05, **p < 0.01, ***p <0.001). n.s., not significant.

TDO2 catalyses the first step of Trp degradation along the KP (37). However, after establishing the regulatory effects of hypoxia on TDO2 expression, we aimed to investigate effects of reduced oxygen on Trp degradation and KP metabolite production, which likely results from both the hypoxia-mediated reduction in TDO2 expression and possibly also reduced TDO2 catalytic activity due to O_2_ limitation. Analysis of supernatants harvested from the long-term hypoxia experiments with A172 cells (shown in Figure 1B), revealed that cells growing under hypoxic conditions produced less Kyn than the cells cultured under normoxia (Figure 2A). This reduction in Kyn production corresponded to the high amount of Trp that remained present in the supernatants of the hypoxic cells (Figure 2B). Taken together, these results indicate that hypoxia downregulates Trp catabolism.

We next hypothesized that the reduced amount of Kyn produced by TDO2 upon hypoxia should affect the metabolic flux of the entire KP. We therefore performed computational modeling of Trp metabolism to predict the steady state fluxes and metabolite concentrations in the KP. To this end, gene expression data from A172 GBM cells cultivated under either hypoxia or normoxia (see Figure 1A) was integrated into the previously mentioned mathematical model of Trp metabolism (29). In line with our hypothesis, the model predicted a significantly decreased Trp catabolic flux mainly caused by the reduced enzymatic flux through TDO2, while the flux through DOPA decarboxylase (DDC) remained unperturbed (Figure 2C). Further, reduced metabolic flux was also predicted for downstream enzymes that degrade Kyn, such as the kynurenine aminotransferases (KATs) and kynurenine-3-monooxygenase (KMO), which generate kynurenic acid (KynA) and 3-hydroxy-anthranilic acid (3HAA), respectively (Figure 2C). This reduced metabolic flux under hypoxia through major enzymes of the KP resulted in reduced simulated intracellular concentrations not only of Kyn but also of its downstream metabolite 3HAA (Figure 2C, gray plots). In contrast, the simulated production of Trp metabolites not directly dependent on TDO2 activity such as tryptamine were predicted to remain unaffected under hypoxia (Figure 2C, gray plots).

To validate the predictions of the mathematical model of Trp metabolism, we analyzed the changes in intracellular concentrations of Trp metabolites in the A172 GBM cells after 5 days of exposure to hypoxia. In line with the simulations, the intracellular concentrations of the KP metabolites Kyn and 3HAA were significantly reduced under hypoxia (Figure 2C, black plots). Although intracellular KynA concentrations were reduced, this decrease failed to attain significance (Figure 2C, black plots). Furthermore, confirming the predictions, the intracellular tryptamine concentrations remained unchanged under hypoxia. In the cell supernatants no changes in 3HAA and KynA levels were observed under hypoxia and tryptamine was undetectable (Supplementary Figure 1). However, in line with our simulations and previous observations, Trp levels in the supernatants increased significantly under hypoxic conditions consistent with the significant decrease in Kyn production (Supplementary Figure 1). Taken together, these results confirm our computational predictions and show that hypoxic downregulation of TDO2 expression reduces flux through the KP.

### TDO2 Expression Is Reduced Upon Stabilization of HIF1α by Chemical Hypoxia Mimetics

**Figure 3:**
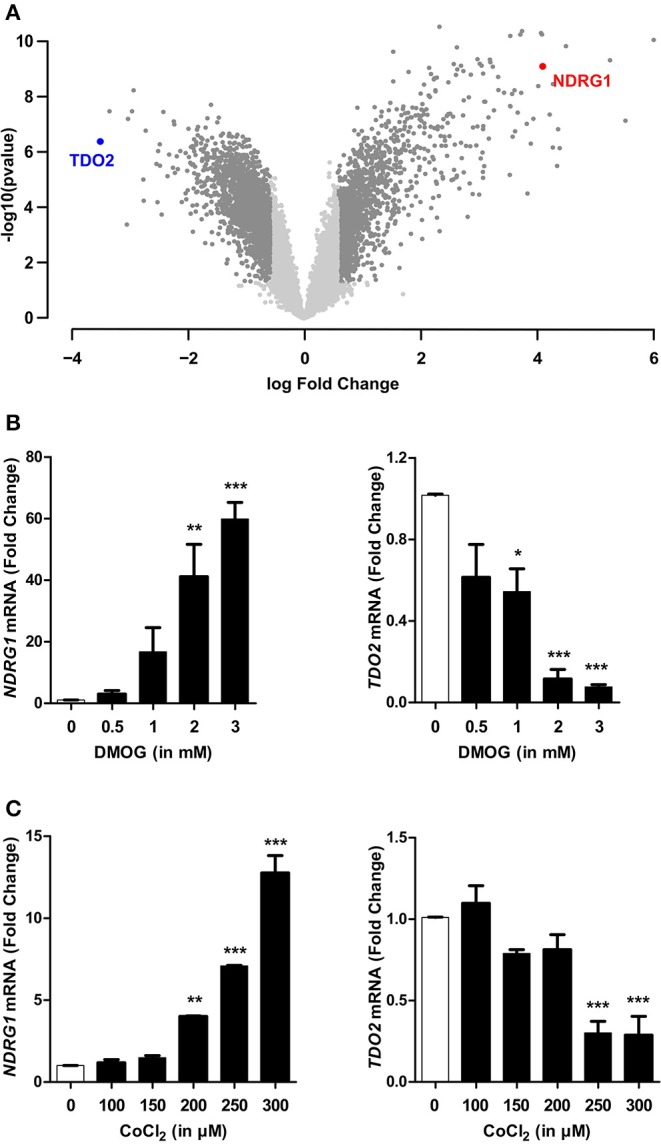
HIF1α stabilization recapitulates hypoxia-mediated reduction in TDO2 expression. **(A)** Volcano plot showing differentially regulated genes in A172 cells exposed to 3 mM DMOG for 24 h compared to DMSO controls. **(B)** qRT-PCR analysis of NDRG1 (left) and TDO2 (right) mRNA expression in A172 cells exposed to 0.5, 1, 2, and 3 mM DMOG for 24 h. **(C)** qRT-PCR analysis of NDRG1 (left) and TDO2 (right) mRNA expression in A172 cells after treatment with 100, 150, 200, 250, and 300 μM of a second HIF1α stabilizing agent, CoCl_2_, for 24 h. Data from at least three independent experiments are expressed as mean ± S.E.M. Statistical significance is assumed at *p* < 0.05 (*p < 0.05, **p < 0.01, ***p < 0.001).

Hypoxia mediates most of its effects through the master regulator HIF1α, however a HIF1α independent global downregulation of gene expression by hypoxia has also been described (4). Therefore, we next investigated if HIF1α plays a role in the hypoxia-mediated downregulation of TDO2. For this, we stabilized HIF1α protein under normoxic conditions by the use of chemical hypoxia mimetics such as dimethyloxalylglycine (DMOG) or cobalt chloride (CoCl_2_). Microarray analysis of A172 cells incubated for 24 h in the presence of 3 mM DMOG (GSE138535), revealed that TDO2 gene expression was strongly downregulated upon HIF1α stabilization (Figure 3A, Supplementary Table 2). qRT-PCR analysis of A172 cells exposed to a range of DMOG concentrations, confirmed the microarray data, as elevated NDRG1 mRNA levels upon DMOG exposure (Figure 3B, left) corresponded to a decrease in TDO2 mRNA expression at tested DMOG concentrations (Figure 3B, right). Analysis of the mRNA expression of A172 cells exposed to a second hypoxia mimetic, cobalt chloride (CoCl_2_), also significantly reduced TDO2 mRNA expression at concentrations of 250 μM CoCl_2_ and above (Figure 3C, right). Correspondingly, mRNA levels of the surrogate hypoxia marker NDRG1 were elevated upon exposure to 200 μM CoCl_2_ and above (Figure 3C, left). In summary, these results indicate that TDO2 expression is regulated by HIF1α.

### siRNA-Mediated Silencing of HIF1α Restores TDO2 Expression

**Figure 4:**
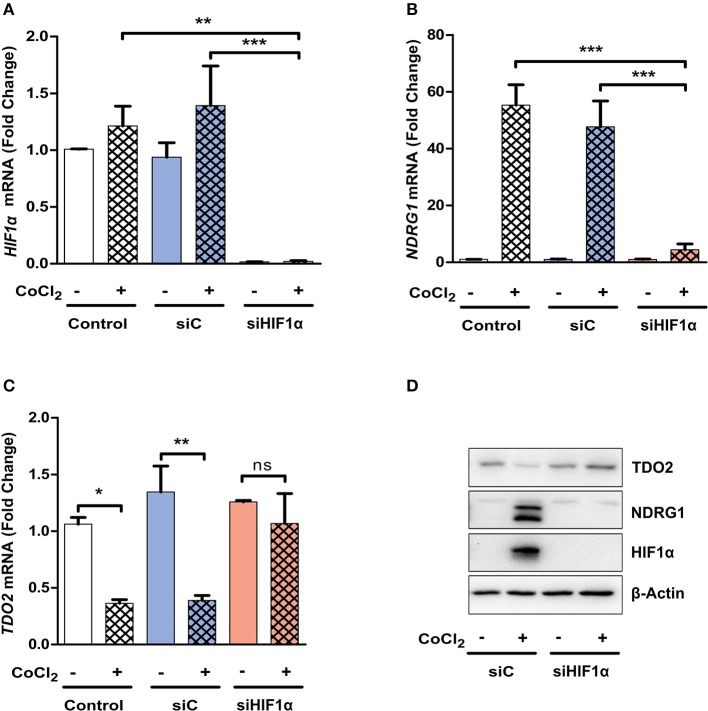
TDO2 expression is rescued upon siRNA-mediated HIF1α silencing. **(A)** qRT-PCR analysis of A172 cells for HIF1α mRNA levels, 24 h post treatment with CoCl_2_ and 48 h post treatments with either non-targeting siC or siRNA targeting HIF1α. **(B)** qRT-PCR analysis of A172 cells as in **(A)**. showing NDRG1 mRNA levels post HIF1α silencing and CoCl_2_ treatment. **(C)** TDO2 mRNA levels analyzed by qRT-PCR in A172 cells treated as in **(A,B)**. **(D)** Western blot analysis of TDO2 and NDRG1 in A172 cells subsequent to siRNA-mediated HIF1α silencing and CoCl_2_ treatment. β-Actin was used as loading control. Data from at least three independent experiments are expressed as mean ± S.E.M. Statistical significance is assumed at *p* < 0.05 (*p < 0.05, **p < 0.01, ***p < 0.001). n.s., not significant.

We next investigated whether the absence of HIF1α can abrogate the observed reduction in TDO2 expression. siRNA-mediated silencing of HIF1α resulted in a significant reduction in HIF1α mRNA (Figure 4A). The reduction of HIF1α was functionally relevant as it prevented the mRNA induction of the surrogate hypoxia marker NDRG1 upon exposure to CoCl_2_ (Figure 4B). Most importantly, however, the suppression of TDO2 mRNA expression upon CoCl_2_ exposure was abrogated in the absence of HIF1α (Figure 4C). Finally, Western blot analysis of A172 cells under the above conditions revealed a complete rescue of TDO2 protein expression upon knockdown of HIF1α (Figure 4D). Taken together, these results confirm that HIF1α controls TDO2 expression in GBM cells.

### Hypoxia Impairs the Ability of Tumor Cells to Suppress T Cell Proliferation

**Figure 5:**
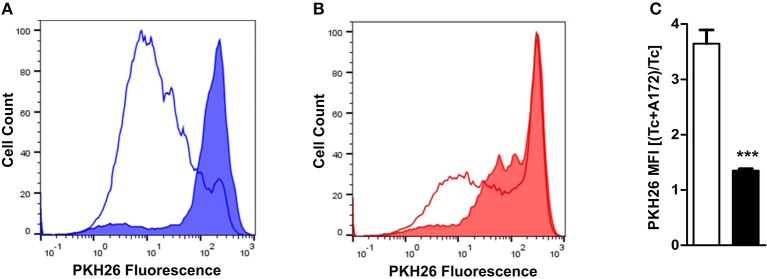
Ability of GBM cells to suppress T cell proliferation is reduced under hypoxia. **(A)** PKH26-labeled CD4^+^ T cells were stimulated and cultured for 6 days either alone or in co-culture with A172 GBM cells. Histogram from a representative experiment where cultures were placed under normoxia (line histogram represents T cells cultured alone, while filled histogram represents T cells co-cultured with A172 cells). **(B)** Experimental setup as described in **(A)**, except that the cells were cultured under hypoxia. **(C)** Plot showing the change in the ratio between the PKH26 mean fluorescence intensity (MFI) of T cells co-cultured with A172 GBM cells vs. those cultured alone, either under normoxia (white) or hypoxia (black). Data from at least three independent experiments are expressed as mean ± S.E.M. Statistical significance is assumed at *p* < 0.05 (***p < 0.001).

TDO2 expression in tumor cells enables them to effectively downregulate the proliferation and thus the anti-tumor activity of infiltrating T cells through production of KP metabolites (32) and the depletion of Trp (38, 39). As our results demonstrate that TDO2 expression is significantly reduced in GBM cells under hypoxic conditions, we next investigated the effect of hypoxia on the proliferation of activated T cells in the presence of A172 GBM cells cultured under either normoxia or hypoxia. Under normoxic conditions, the GBM cells in the co-culture system were clearly capable of suppressing T cell proliferation as compared to the normoxic T cell mono-cultures (Figure 5A). However, under hypoxic conditions the previously observed T cell suppression by GBM cells was reduced (Figure 5B). Quantification of T cell proliferation expressed as PKH26 mean fluorescent intensity (MFI) revealed that exposure to hypoxia significantly reduced the T cell suppressive capacity of A172 GBM cells in the co-culture system (Figure 5C).

## Discussion

Over the past decades, the role of hypoxia in shaping the tumor microenvironment and its contribution to tumor cell intrinsic properties as well as anti-tumor immunity has been well-documented (17). Hypoxia is a frequently occurring feature in most solid tumors including GBM, where it not only drives tumor malignancy but also determines tumor morphology (8).

Here, we set out to investigate the role of hypoxia in controlling GBM-derived factors that impact anti-tumor immune responses. Gene expression analysis identified TDO2 as the immunomodulatory factor most strongly regulated in response to hypoxia. TDO2 is a heme containing dioxygenase enzyme, which catalyses the first step of the KP, namely the conversion of Trp to formyl-kynurenine (32). Trp is the least abundant essential amino acid in humans, which in addition to its role in protein synthesis also functions as the precursor for diverse neurotransmitters, hormones and vitamins including serotonin, tryptamine, melatonin, and nicotinamide (40, 41). Trp catabolism along the KP is a well-known modulator of immune responses. Initially identified as an immunosuppressive mechanism preventing the rejection of allogeneic fetuses (42), Trp catabolism has also been implicated in neuropsychiatric disorders (43, 44), auto-immune and inflammatory diseases (45, 46).

Moreover, human cancers often express high levels of indoleamine-2,3-dioxygenase 1 (IDO1) and/or TDO2, the initial Trp-catabolic enzymes of the KP (37). TDO2, for instance is expressed in diverse tumor entities including breast cancer, bladder cancer, hepatocellular carcinoma, melanoma, non-small cell lung cancer, ovarian carcinoma, renal cell carcinoma, and GBM, where it promotes tumor cell motility and suppresses T cell proliferation and function (24, 47, 48). As Trp catabolism along the KP plays an important tumor-promoting role, this has resulted in interest toward targeting the enzymes of this pathway for cancer therapy (49).

Abnormal or inadequate vasculature in GBM results in formation of regions that have restricted nutrient and oxygen supply (50). Malignant cells in these nutrient-deprived hypoxic regions adapt to survive by profound metabolic reprogramming. In human GBM cells, numerous genes involved in global cellular metabolism are downregulated in response to hypoxia (15). This enables the cells to conserve nutrients in order to redirect them toward essential life-sustaining processes. Hypoxic regions in GBM, due to their nutrient-restricted microenvironment, tend to have a limited supply of Trp, which would dictate that cells conserve Trp under hypoxia. Here, we show that indeed upon hypoxic exposure GBM cells reversibly downregulate TDO2 expression (Figures 1A–E), which is restored upon availability of oxygen (Figure 1F). This reversibility may enable tumor cells to effectively regulate Trp catabolism as necessary under cyclic hypoxia, which has been described to frequently occur during tumor progression and metastasis (51). In line with the downregulation of TDO2, the amount of downstream Kyn produced under hypoxia was reduced significantly (Figure 2A), corresponding to higher levels of Trp remaining in the extracellular space (Figure 2B).

In humans, Trp can be degraded by a number of enzymes along different metabolic pathways, however a majority of the available free Trp has been reported to be degraded via the KP (37). Therefore, we hypothesized that hypoxic control of TDO2 expression, might influence global Trp flux in a tumor cell. We employed a previously described computational model (29) to integrate gene expression data in order to predict changes in Trp metabolism under hypoxia. Our predictions revealed that indeed under hypoxia, the global Trp flux was significantly reduced (Figure 2C, blue plots). This reduction upon hypoxia can be attributed to the reduction in metabolic flux through TDO2 and consequently other downstream enzymes in the KP (Figure 2C, blue plots). In line, the computational model predicted that intracellular concentrations of Kyn and its downstream metabolite 3HAA were significantly reduced under hypoxia (Figure 2C, gray plots). In contrast, the flux through enzymes outside the KP, such as DDC, which degrades Trp to the neuromodulator tryptamine, remained virtually unperturbed (Figure 2C, blue plots). These predictions substantiate our hypothesis, that under hypoxia tumor cells downregulate TDO2 expression in order to conserve Trp.

To validate the computational predictions, we next measured intracellular metabolite concentrations of A172 GBM cells cultured under the same conditions as for the microarray analysis. The measured intracellular concentrations of Kyn and other Trp metabolites reflected the exact pattern of the predicted concentrations (Figure 2C, black plots), demonstrating that prediction of Trp metabolite concentrations accurately reflects their relative changes. The measurements showed that under hypoxic conditions the intracellular pool of Trp remains largely unchanged due to a significant reduction in the flux through TDO2. The latter was reflected by a reduced production of Kyn and downstream metabolites, while the concentration of other Trp metabolites such as tryptamine was unaltered (Figure 2C). Previously, we have shown that tumor cells in a nutrient-deficient but normoxic microenvironment upregulate the expression of tryptophanyl-tRNA synthetase to better utilize the available Trp pool for protein synthesis (52). Taken together, our current results establish the presence of a second adaptation to limited Trp availability under nutrient stress, where tumor cells conserve Trp by downregulation of TDO2 under hypoxic conditions.

Reduced Kyn levels in response to hypoxia have previously been attributed in tumor cells and fibroblasts to reduced expression and activity of IDO1 (53–55), which catalyzes the same reaction as TDO2. The presence of chemokines or chemokine-producing immune cells can however increase the expression of IDO1 under hypoxia (54, 56). Other studies have also reported the upregulation of IDO1 expression upon hypoxic exposure or HIF1α stabilization in neural and immune cells (57–59). Taken together, these studies indicate that the regulation of IDO1 under hypoxic conditions is highly cell type specific and also depends upon microenvironmental factors such as immune cell infiltration. Elbers and colleagues also previously described hypoxia-mediated downregulation of Trp metabolism, which they attributed to reduced TDO2 enzymatic activity under hypoxia (60). The authors used recombinant TDO2 protein in an overexpression system to arrive at the aforementioned conclusions. However, our results provide evidence for the existence of a transcriptional mechanism regulating TDO2-mediated Trp degradation under hypoxia.

Most biological effects of TDO2 including its immunosuppressive actions can be attributed either to the depletion of Trp, which activates nutrient sensing mechanisms such as GCN2 (61) or to the accumulation of downstream KP metabolites. The KP metabolite 3HAA modulates immune functions by enhancing the differentiation of Tregs, reducing T cell proliferation and inducing T cell death (62–64). 3HAA can also interfere with the anti-tumor activity of macrophages by inhibiting their NO production (64). Moreover, 3HAA can be converted further along the KP to quinolinic acid (QA) (65), which can serve as a precursor for NAD+ biosynthesis (66–68). In line with immunosuppressive effects of 3HAA, its metabolic product QA can also modulate immune cell function by suppressing T cell proliferation and increasing Tregs (64).

AHR activation accounts for many of the effects of Trp degradation (24, 69–72). KP metabolites including Kyn, kynurenic acid, xanthurenic acid, and cinnabarinic acid are potent AHR activators (24, 69–72). Moreover, engagement of nuclear coactivator 7 by 3HAA has been reported to enhance activation of the AHR in dendritic cells (73). AHR activation results in AHR binding to HIF1β (ARNT), which also is a binding partner for HIF1α upon its hypoxia-mediated stabilization. Sharing a common binding partner increases the likelihood of competition between the two transcription factors for ARNT binding in scenarios where both AHR activation and HIF1α stabilization take place. In line, reports indicate that HIF1α stabilization adversely affects the activity and downstream gene regulation subsequent to AHR activation in an ARNT-dependent fashion (74). Thus, hypoxia could counteract TDO2 effects mediated via AHR. Furthermore, in GBM, signaling through HIF1α and AHR can crossregulate each other at several points of contact, coordinating metabolic regulation of anti-tumor immunity as well as tumor growth (75).

In this light, we investigated if HIF1α can also interfere upstream of AHR by regulating TDO2 expression or whether the observed hypoxia-mediated TDO2 downregulation is a general HIF1α- independent hypoxia effect. We used the hypoxia mimetic DMOG to stabilize HIF1α in A172 GBM cells under normoxic conditions. Microarray results and qRT-PCR measurements identified TDO2 to be the most downregulated gene upon HIF1α stabilization by DMOG (Figures 3A,B). Further, use of a second HIF1α stabilizing agent, CoCl_2_, also resulted in a significant reduction in TDO2 expression (Figure 3C). siRNA-mediated knockdown of HIF1α rescued TDO2 expression (Figures 4A–D). Taken together, these results suggest that HIF1α employs a two-pronged strategy to regulate AHR activity, first by direct competitive binding of ARNT (74) and second by downregulating TDO2 expression, thus reducing the concentration of AHR-activating Trp-metabolites.

Although lower oxygen levels are essential for immune cell maturation, extreme pathological hypoxia especially in a tumor acts as an effective immunosuppressive strategy, helping tumors escape immune surveillance (20). TDO2 also helps tumor immune evasion by activating the AHR through its downstream metabolites (24). In light of the existence of these two distinct modi operandi of tumors to suppress anti-tumor immunity, we next ascertained their role in tumor immune suppression under hypoxic conditions. Our data revealed a significant reduction in the immune suppressive abilities of GBM cells in hypoxic co-cultures with T cells (Figures 5A–C).

In the present study, we report a HIF1α-dependent regulatory mechanism in GBM cells through which hypoxia can reversibly regulate the expression of the Trp-degrading enzyme TDO2 and thus the production of known immunosuppressive onco-metabolites. Our results further suggest that GBM cells in their quest to give anti-tumor immunity a slip, employ the immunosuppressive effects of both TDO2 and hypoxia in a well-coordinated fashion. In microenvironments with ample oxygen and nutrient availability, tumor cells can employ the TDO2-Kyn-AHR axis to suppress the immune system. While in a nutrient-deficient hypoxic microenvironment, where hypoxia itself keeps the immune system in check, tumor cells in a HIF1α-dependent fashion can downregulate TDO2 expression so as to conserve Trp. This novel mechanism may present new insights for better clinical management of anti-tumor immune suppression by both TDO2 expression as well as by hypoxia.

## Data Availability Statement

The expression datasets generated in this study are deposited in the Gene Expression Omnibus (GEO) respository under the accession number GSE138535.

## Ethics Statement

The studies involving human participants were reviewed and approved by the Ethics Committee of the University of Heidelberg. Written informed consent for participation was not required for this study in accordance with the national legislation and the institutional requirements.

## Author Contributions

SM and CO designed the study and wrote the manuscript. SM, L-OT, GP, and CO developed the methodology. SM, L-OT, and GP acquired the data. SM, AS, L-OT, JD, GP, IH, and CO analyzed and interpreted the data. All the authors read, reviewed, and revised the manuscript.

### Conflict of Interest

The authors declare that the research was conducted in the absence of any commercial or financial relationships that could be construed as a potential conflict of interest.

## Supplementary Material

Supplementary Table 1 Top differentially regulated genes in A172 GBM cells post exposure to 5 days of hypoxia.

Supplementary Table 2 Top differentially regulated genes in A172 GBM cells 24 h post-exposure to 3 mM DMOG.

Supplementary Figure 1 Metabolic measurements of supernatants from A172 GBM cells cultured for 5 days either under normoxia (white) or hypoxia (black). **(A)** Trpyptophan (Trp), **(B)** Kynurenine (Kyn), **(C)** 3-hydroxyanthranilic acid (3HAA), **(D)** Kynurenic acid (KynA), **(E)** Tryptamine (Trypta). Data from at least three independent experiments are expressed as mean ± S.E.M. Statistical significance is assumed at *p* < 0.05 (****p* < 0.001, *****p* ≤ 0.0001). n.s., not significant and n.d., not detected.
