# Supplementary material for: Hypoxia Inducible Factor 1α Inhibits the Expression of Immunosuppressive Tryptophan-2,3-Dioxygenase in Glioblastoma
Source: Front Immunol. 2019 Dec 4;10:2762. doi: 10.3389/fimmu.2019.02762 (PMC6905408; doi:10.3389/fimmu.2019.02762)
Supplement: Supplementary Table 1 — Top differentially regulated genes in A172 GBM cells post exposure to 5 days of hypoxia. [file Table_1.docx]

**SUPPLEMENTARY TABLE 1**

Top differentially regulated genes in A172 GBM cells post exposure to 5 days of hypoxia.

| **Top 10 Upregulated Genes** | | **Top 10 Downregulated Genes** | |
| --- | --- | --- | --- |
| **Gene Symbol** | **Gene Name** | **Gene Symbol** | **Gene Name** |
| EGLN3 | Egl-9 Family Hypoxia Inducible Factor 3 | TAF9B | TATA-Box Binding Protein Associated Factor 9b |
| NDRG1 | N-Myc Downstream Regulated 1 | TDO2 | Tryptophan 2,3-Dioxygenase |
| ALDOC | Aldolase, Fructose-Bisphosphate C | TLR3 | Toll Like Receptor 3 |
| STC1 | Stanniocalcin 1 | PHACTR1 | Phosphatase And Actin Regulator 1 |
| DEPP1 | DEPP1 Autophagy Regulator | RGS16 | Regulator Of G Protein Signaling 16 |
| UCA1 | Urothelial Cancer Associated 1 | DEPTOR | DEP Domain Containing MTOR Interacting Protein |
| PDK1 | Pyruvate Dehydrogenase Kinase 1 | SPTLC3 | Serine Palmitoyltransferase Long Chain Base Subunit 3 |
| RNASE4 | Ribonuclease A Family Member 4 | SELENOP | Selenoprotein P |
| ENO2 | Enolase 2 | CCL2 | C-C Motif Chemokine Ligand 2 |
| IGFBP3 | Insulin Like Growth Factor Binding Protein 3 | PIK3R3 | Phosphoinositide-3-Kinase Regulatory Subunit 3 |

**SUPPLEMENTARY TABLE 2**

Top differentially regulated genes in A172 GBM cells 24 h post-exposure to 3 mM DMOG.

| **Top 10 Upregulated Genes** | | **Top 10 Downregulated Genes** | |
| --- | --- | --- | --- |
| **Gene Symbol** | **Gene Name** | **Gene Symbol** | **Gene Name** |
| DEPP1 | Decidual Protein Induced By Progesterone | TDO2 | Tryptophan 2,3-Dioxygenase |
| RORA | RAR Related Orphan Receptor A | FAM84B | Family With Sequence Similarity 84 Member B |
| ANGPTL4 | Angiopoietin Like 4 | ZSCAN31 | Zinc Finger And SCAN Domain Containing 31 |
| DDIT4 | DNA Damage Inducible Transcript 4 | CD70 | Tumor Necrosis Factor Ligand Superfamily Member 7 |
| ZP1 | Zona Pellucida Glycoprotein 1 | HNMT | Histamine N-Methyltransferase |
| SH2D1B | SH2 Domain Containing 1B | DIRAS3 | DIRAS Family GTPase 3 |
| HK2 | Hexokinase 2 | ETV1 | ETS Variant 1 |
| EFCAB3 | EF-Hand Calcium Binding Domain 3 | OAS1 | 2'-5'-Oligoadenylate Synthetase 1 |
| RIMKLA | N-Acetylaspartylglutamate Synthase A | LRIG3 | Leucine Rich Repeats And Immunoglobulin Like Domains 3 |
| NDRG1 | N-Myc Downstream Regulated 1 | FAM198B | Family With Sequence Similarity 198, Member B |
