# Supplementary figures and images for: Hypoxia Inducible Factor 1α Inhibits the Expression of Immunosuppressive Tryptophan-2,3-Dioxygenase in Glioblastoma
Source: Front Immunol. 2019 Dec 4;10:2762. doi: 10.3389/fimmu.2019.02762 (PMC6905408; doi:10.3389/fimmu.2019.02762)

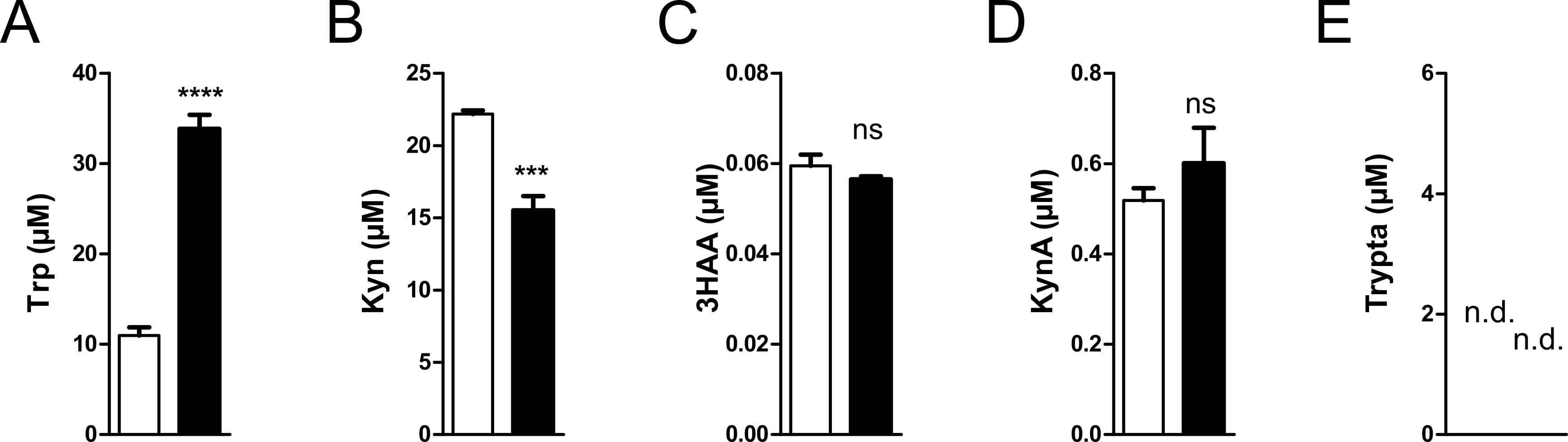

Supplement: Supplementary Figure 1 — Metabolic measurements of supernatants from A172 GBM cells cultured for 5 days either under normoxia (white) or hypoxia (black). (A) Trpyptophan (Trp), (B) Kynurenine (Kyn), (C) 3-hydroxyanthranilic acid (3HAA), (D) Kynurenic acid (KynA), (E) Tryptamine (Trypta). Data from at least three independent experiments are expressed as mean ± S.E.M. Statistical significance is assumed at p < 0.05 (***p < 0.001, ****p ≤ 0.0001). n.s., not significant and n.d., not detected. [file Image_1.JPEG]
